# An Analysis of 50 Cases of Salivary Gland Neoplasms: A Single-Institution Experience

**DOI:** 10.7759/cureus.103480

**Published:** 2026-02-12

**Authors:** Kanwalpreet Kaur, Gurbax Singh, Sumit Prinja, Sarita Nibhoria

**Affiliations:** 1 Otolaryngology - Head and Neck Surgery, Guru Gobind Singh Medical College, Faridkot, IND; 2 Otolaryngology - Head and Neck Surgery, Government Medical College, Amritsar, IND; 3 Pathology, Guru Gobind Singh Medical College, Faridkot, IND

**Keywords:** adenoid cystic carcinoma, mucoepidermoid carcinoma, parotidectomy, pleomorphic adenoma, salivary gland neoplasms

## Abstract

Aim

Salivary gland neoplasms represent a rare subset of head and neck neoplasms. Due to their varied histological and biological behaviours, they pose a challenge for both surgeons and histopathologists. The study aimed to describe the demographic and clinical characteristics of patients with salivary gland neoplasms at our centre, assess their anatomical distribution and histopathological patterns, and evaluate the concordance of fine needle aspiration cytology (FNAC) with histopathology. The study also documented surgical management approaches, short-term postoperative outcomes, including procedure-specific complications, and the use of adjuvant therapy.

Materials and methods

A prospective descriptive study was conducted at a single institution for 18 months on 50 consecutive patients diagnosed with primary salivary gland neoplasms. Data were collected by documenting clinical history and performing physical examinations and investigations. After surgery, depending on the histopathological examination reports, radiotherapy was considered. Patients were monitored for three months after surgery. Statistical analyses were conducted using SPSS version 22.0 (IBM Corp., Armonk, NY). Group differences were examined using the chi-square test, with significance set at p<0.05.

Results

In our study of 50 neoplasms, 38 were classified as benign and 12 as malignant. The 5th decade observed the highest incidence of benign neoplasms, while most reports of malignant neoplasms came from the 4th to 6th decades. In the present cohort, a female preponderance was observed in pleomorphic adenoma, whereas mucoepidermoid carcinoma showed male predominance. FNAC and histopathology agreed in 43 cases, giving an overall diagnostic accuracy of 86.0% and Cohen’s k of ≈0.55 (moderate agreement). In a benign-malignant comparison, FNAC had 100% sensitivity for benign lesions but low specificity (41.7%), indicating reliable detection of benign neoplasms but limited ability to exclude malignancy. Surgery was the main treatment modality in all cases, whereas adjuvant therapy (radiotherapy) was administered in 24% of cases (n=12).

Conclusion

The majority of salivary gland neoplasms were benign. Benign neoplasms tended to be more common in females, whereas malignant neoplasms were predominantly observed among males. Pleomorphic adenoma was most prevalent in the parotid gland, and mucoepidermoid carcinoma was most frequently diagnosed in the submandibular gland.

## Introduction

Salivary gland neoplasms are uncommon, accounting for 3%-6% of head and neck neoplasms and about 0.3%-0.5% of all malignancies [1]. Epidemiological studies show an annual incidence from 0.4 to 13.5 cases per 100,000 persons [2,3]. The highest occurrence is during the 3rd and 4th decades for benign neoplasms and the 4th and 5th decades for malignant neoplasms [4]. 35% of these neoplasms are diagnosed as malignant in the pediatric population [5]. The majority of cases occur in adults, with benign lesions most frequently observed in middle-aged females [6]. They also exhibit a female predominance [7-9]; however, this pattern is not universal [10].

The parotid gland accounts for two-thirds to four-fifths of these neoplasms, while about 10% arise in the submandibular gland and the remainder in the sublingual and minor salivary glands [11]. The likelihood that a salivary gland neoplasm is malignant is inversely proportional to gland size because larger glands (especially the parotid) are predominantly serous, well-encapsulated, and allow slow-growing benign neoplasms, whereas smaller and minor salivary glands have more mucous cells, less stromal protection, greater environmental exposure, and limited space, making neoplasms more likely to be aggressive and malignant [12,13].

The benign-malignant distribution of salivary gland neoplasms varies by site, with parotid neoplasms being mostly benign neoplasms and minor salivary gland neoplasms being predominantly malignant [14]. Most minor salivary gland neoplasms originate on the palate [10], with pleomorphic adenoma and mucoepidermoid carcinoma being the predominant benign and malignant types, respectively [15].

These neoplasms exhibit complex clinicopathological characteristics and distinct biological behaviours [16]; therefore, the World Health Organization (WHO) revised its classification in the 5th edition (2022) to promote diagnostic standardisation, inform treatment strategies, and aid in prognostic assessment [17].

Benign salivary gland neoplasms typically present as slow-growing, painless, well-circumscribed, mobile masses with normal overlying skin or mucosa and intact facial nerve function. In contrast, malignant neoplasms often show rapid growth, pain, ill-defined and fixed margins, ulceration, facial nerve involvement, regional lymphadenopathy, perineural invasion, and features of local invasion or distant metastasis [18]. The principal intervention involves surgery. Malignant neoplasms pose a significant challenge in treatment, as local invasion increases the likelihood of leaving behind islands of the neoplasm during surgery, thereby leading to recurrences [19]. Benign parotid neoplasms are usually treated with superficial or total conservative parotidectomy, preserving the facial nerve, whereas malignant parotid lesions require total parotidectomy, with nerve sacrifice if involved, and neck dissection for high-grade or node-positive disease [20].

Given the higher malignant potential of submandibular gland neoplasms compared with parotid tumours, complete excision of the gland is favoured even when lesions appear clinically benign, to minimise the risk of under-treating occult malignancy [21]. Sublingual gland neoplasms are uncommon but predominantly malignant; therefore, treatment generally involves wide en bloc resection, with selective neck dissection considered in cases with high-risk histology or advanced stage [22]. Minor salivary gland neoplasms, commonly involving the palate, are frequently malignant and are managed by wide local excision with adequate margins. Advanced lesions may necessitate mandibulectomy or maxillectomy, depending on the extent of local invasion [18].

Parotidectomy may be associated with early complications such as salivary fistula, sialocele, hematoma, sensory loss, wound infection, and cosmetic deformity, while delayed sequelae, including Frey’s syndrome and first bite syndrome, can adversely affect long-term quality of life [23]. Transient facial nerve dysfunction has been reported in approximately 45%-60% of patients undergoing total parotidectomy, whereas rates after superficial parotidectomy are considerably lower, ranging from 10% to 25% [24]. Permanent facial nerve deficits are uncommon overall but occur more frequently after total parotidectomy, particularly in cases involving deep lobe lesions or extensive dissection [25]. Owing to the proximity of the marginal mandibular, lingual, and hypoglossal nerves, postoperative complications of submandibular gland excision may include transient or permanent nerve dysfunction, hematoma, infection, sensory disturbances, and scarring [26].

## Materials and methods

Study settings

The study was undertaken in the Department of Otorhinolaryngology at Guru Gobind Singh Medical College and Hospital, Faridkot.

Study period

The total study duration was 18 months, including patient recruitment, surgery, and postoperative follow-up.

Study design

A prospective descriptive observational study design was employed.

Study population

The study included patients of all age groups presenting to the otorhinolaryngology outpatient department with salivary gland swellings that met the specified inclusion criteria.

Inclusion criteria

Patients with swellings arising from the salivary glands that proved to be neoplastic were included in the study.

Exclusion criteria

Patients with congenital cysts and masses, chronic sialadenitis, sialolithiasis, granulomatous diseases, Sjogren’s syndrome, and secondary invasion of salivary glands by cutaneous malignancies were excluded.

Sample size

The study comprised 50 consecutively selected patients.

Sampling technique

A non-probability consecutive sampling method was used.

Methodology

 A pre-designed structured proforma was utilised to record each patient’s medical history, clinical examination, and results of diagnostic investigations.

The clinical history included demographic details (name, age, gender, occupation, and address), along with presenting complaints, duration of illness, medical history, and any prior treatment for the current condition.

The initial evaluation comprised a comprehensive physical examination followed by a targeted regional assessment. A thorough head and neck examination was conducted. Swelling was evaluated for site, number, size, extent, consistency, mobility, and neurological involvement. The regional lymphatic regions were palpated to evaluate nodal count, size, site, mobility, and fixation to the overlying skin.

The baseline diagnostic workup included a complete blood count, viral serologies, coagulation studies, random blood glucose, serum electrolyte assessment, hepatic and renal function tests, electrocardiography, and a chest radiograph in the posteroanterior view. The cytomorphological characteristics of each lesion were assessed by fine needle aspiration cytology (FNAC) and classified according to the Milan System for Reporting Salivary Gland Cytopathology, which defines malignancy risk and guides management. Ultrasonography of the neck aided in the detection, localisation, and characterisation of lesions, and in evaluating regional lymph node involvement. The excised specimens were processed and examined histopathologically to establish the definitive diagnosis.

Surgical management constituted the principal therapeutic modality for these neoplasms. Superficial parotidectomy with meticulous facial nerve preservation was undertaken for benign parotid gland neoplasms and also for small malignant neoplasms limited to the superficial lobe. Total parotidectomy was performed in deep lobe parotid neoplasms to achieve adequate neoplasm clearance. Management of submandibular gland neoplasms was achieved through complete glandular excision. Minor salivary gland neoplasms were treated by wide local excision with sufficient margins to ensure complete removal of the lesion. An endoscopic approach was utilised for the resection of maxillary sinus neoplasms. Excision of parapharyngeal space neoplasms was performed via a transcervical approach. Postoperative radiotherapy was used in patients with high-grade malignancies, deep lobe parotid neoplasms, and enlarged cervical lymph nodes.

A postoperative follow-up of three months was conducted for all patients to check for any potential complications, including facial nerve paralysis, haemorrhage, infection, skin flap necrosis, trismus, sialocele, seroma, Frey’s syndrome, injury to the marginal mandibular branch of the facial nerve, lingual nerve injury, hypoglossal nerve injury, fistula formation, xerostomia, and neoplasm recurrence.

Statistical analysis

Data were entered into Microsoft Excel (Microsoft Corp., Redmond, WA, USA) and analysed using Statistical Package for the Social Sciences (SPSS) version 22.0 (IBM Corp., Armonk, NY). Continuous variables were summarised as means and standard deviations, while categorical variables were expressed as frequencies and percentages. Associations between categorical variables were analysed using the chi-square test when the assumption of adequate expected cell frequencies (≥5 in at least 80% of cells) was satisfied. In cases where expected cell counts were insufficient, Fisher’s exact test was applied. A p-value of < 0.05 was considered statistically significant. The diagnostic performance of FNAC was evaluated by comparison with histopathological examination, which served as the reference standard. Diagnostic accuracy was assessed by calculating sensitivity, specificity, positive predictive value, negative predictive value, and overall concordance. Cases in which FNAC and histopathological diagnoses were discordant were classified as false-positive or false-negative findings and included in the final analysis.

Consent

The patients were provided with a consent form to obtain their approval. The study's purpose was briefed to them in their native language. Only those who gave written and informed consent were included in the study. Participation was entirely voluntary, and patients could withdraw at any time.

Ethical considerations 

(1) Approval of the ethical committee was obtained. (2) Informed and written consent was ensured. (3) Strict confidentiality of data was maintained.

## Results

The study included 50 patients diagnosed with primary salivary gland neoplasms. Patient ages ranged from 1 to 80 years. The median age was 45 years (interquartile range, IQR: 30.5-54 years). The mean ± standard deviation age was 42.3 ± 17.15 years. The mean ± standard deviation ages of males and females during the period of diagnosis were 37.9 ± 17.64 and 45.48 ± 16.36 years, respectively.

The mean ± standard deviation age was 40.5 ± 18.6 years for pleomorphic adenoma, 45.8 ± 8.3 years for mucoepidermoid carcinoma, 42.3 ± 10.8 years for adenoid cystic carcinoma, and 50.0 ± 1.4 years for squamous cell carcinoma. The single cases of salivary duct carcinoma and lymphoepithelial carcinoma occurred at ages 60 and 65 years, respectively.

**Figure 1:**
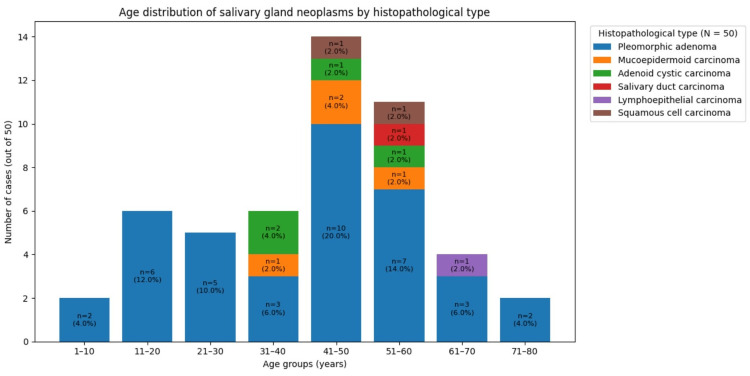
Age distribution based on histopathological types of salivary gland neoplasms N = total cases, while n = cases within each category

The median age of patients with benign neoplasms was 42 years (IQR: 20-55), while that of patients with malignant neoplasms was 49.5 years (IQR: 35-53). A two-tailed Mann-Whitney U test showed no statistically significant difference in age between the two groups (U=143, p=0.25). Although benign neoplasms were most frequent in the 5th decade and malignant neoplasms in the 4th-6th decades, this difference did not reach statistical significance. Pleomorphic adenoma and mucoepidermoid carcinoma were frequent in patients in their forties, while adenoid cystic carcinoma predominated in the thirties (Figure 1). 

Females constituted the majority of cases overall (n=29, 58%). Benign neoplasms were more frequently observed in females (n=24, 63.16%), whereas malignant neoplasms were more commonly observed in males (n=7, 58.33%). A higher proportion of pleomorphic adenoma cases occurred in females, whereas mucoepidermoid carcinoma cases were more often observed in males, as depicted in Table 1. This association was not statistically significant (chi-square=1.21, p=0.28) and is therefore presented as a descriptive pattern. The odds of malignancy were lower in females compared with males (odds ratio=0.42; 95% CI: 0.11-1.57); however, the wide confidence interval reflects limited precision due to the small number of malignant cases (n=12). Fisher’s exact test was applied when expected cell counts were fewer than five to ensure the validity of categorical comparisons.

**Table 1:** Gender distribution based on histopathological types of salivary gland neoplasms n = number of cases

Histopathological types of neoplasms	Male		Female		Total	
	n	%	N	%	n	%
Pleomorphic adenoma	14	66.67	24	82.76	38	76
Mucoepidermoid carcinoma	3	14.29	1	3.45	4	8
Adenoid cystic carcinoma	2	9.52	2	6.90	4	8
Salivary duct carcinoma	1	4.76	-	-	1	2
Lymphoepithelial carcinoma	-	-	1	3.45	1	2
Squamous cell carcinoma	1	4.76	1	3.45	2	4
Total	21	100	29	100	50	100
Chi-square	1.21					
p-value	0.28					

Table 2 shows that benign neoplasms were most often found in the parotid gland, while the malignant neoplasms occurred most often in the submandibular gland (7/12, 58%; 95% CI: 27%-84%), followed by the parotid gland (2/12, 17%; 95% CI: 2%-48%), maxillary sinus (2/12, 17%; 95% CI: 2%-48%), and parapharyngeal space (1/12, 8%; 95% CI: 0%-38%). Adenoid cystic carcinoma was equally distributed between the submandibular gland (n=2) and the maxillary sinus (n=2).

**Table 2:** Frequency of the salivary gland neoplasms according to site

Histopathological types of neoplasms	Parotid gland	Submandibular gland	Hard palate	Parapharyngeal space	Maxillary sinus	Lip
Pleomorphic adenoma	24	10	2	1	-	1
Mucoepidermoid carcinoma	-	3	1	-	-	-
Adenoid cystic carcinoma	-	2	-	-	2	-
Salivary duct carcinoma	1	-	-	-	-	-
Lymphoepithelial carcinoma	1	-	-	-	-	-
Squamous cell carcinoma	-	2	-	-	-	-
Total	26	17	3	1	2	1

Table 3 summarises the presenting features of salivary gland neoplasms. Swelling was the most common symptom in all cases, while tenderness and skin fixity were each observed in only 1 of 12 malignant cases (8.3%) and were absent in benign cases. These findings were purely descriptive.

**Table 3:** Clinical features of salivary gland neoplasms n = number of cases

Symptom	Benign (n=38)	Malignant (n=12)
Swelling	38/38 (100%)	12/12 (100%)
Tenderness	0/38 (0%)	1/12 (8.3%)
Skin fixity	0/38 (0%)	1/12 (8.3%)

The diagnostic efficacy of FNAC, with histopathology as the gold standard, is portrayed in Table 4. FNAC showed an overall accuracy of 86.0% (43/50), with a Cohen’s κ of approximately 0.55, indicating moderate agreement with histopathological diagnosis. The high accuracy observed for less frequent malignancies largely reflects a high proportion of true negatives and small case numbers and should therefore be interpreted as descriptive.

**Table 4:** Correlation of fine needle aspiration cytology findings with histopathological diagnosis across different salivary gland neoplasms *Denotes “∞/undefined” values for LR+ and DOR, which occur when specificity equals 1.000 (no false positives), leading to division by zero. These reflect perfect specificity in this sample and should be interpreted with caution due to small subgroup counts.

Histopathological types of neoplasms	Pleomorphic adenoma	Mucoepidermoid carcinoma	Adenoid cystic carcinoma	Salivary duct carcinoma	Lymphoepithelial carcinoma	Squamous cell carcinoma
True positive	38	3	1	0	0	1
False positive	7	0	0	0	0	0
False negative	0	1	3	1	1	1
True negative	5	46	46	49	49	48
Sensitivity	1.00 (100.0%)	0.75 (75.0%)	0.25 (25.0%)	0.00 (0.0%)	0.00 (0.0%)	0.50 (50.0%)
95% CI (Clopper–Pearson)	0.907-1.000	0.194-0.994	0.006-0.806	0.000-0.975	0.000-0.975	0.013-0.987
Specificity	0.4167 (41.7%)	1.000 (100.0%)	1.000 (100.0%)	1.000 (100.0%)	1.000 (100.0%)	1.000 (100.0%)
95% CI (Clopper–Pearson)	0.152-0.723	0.923-1.00	0.923-1.00	0.927-1.00	0.927-1.00	0.926-1.00
Positive predictive value	0.8444 (84.4%)	1.000 (100.0%)	1.000 (100.0%)	- (no positives)	- (no positives)	1.000 (100.0%)
Negative predictive value	1.000 (100.0%)	0.9787 (97.9%)	0.9388 (93.9%)	0.98 (98.0%)	0.98 (98.0%)	0.9787 (97.9%)
Positive likelihood ratio	81.7143	∞ (undefined)*	∞ (undefined)*	-	-	∞ (undefined)*
Negative likelihood ratio	0.0	0.25	0.75	1.00	1.00	0.50
Diagnostic odds ratio	∞ (undefined)*	∞ (undefined)*	∞ (undefined)*	-	-	∞ (undefined)*
Diagnostic accuracy	86%	98%	94%	98%	98%	98%

**Figure 2:**
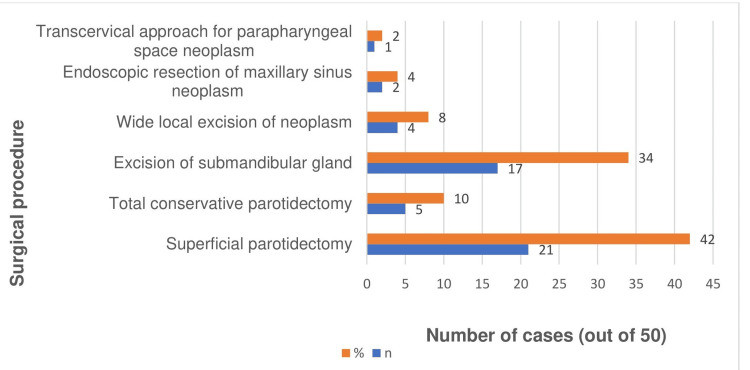
Surgical procedures performed, shown as absolute numbers (n) and percentages (%) of the total cohort (N=50)

Surgery was the primary modality of treatment. According to Figure 2, superficial parotidectomy (nerve sparing) was the most common procedure performed. 

Adjuvant radiotherapy was administered to 12 patients with malignant neoplasms. Table 5 details neoplasms' sites, histology, margin status, perineural invasion, nodal involvement, and indication for radiation. The most frequently irradiated site was the submandibular gland (n=7), followed by the parotid gland (n=2), maxillary sinus (n=2), and hard palate (n=1).

**Table 5:** Details of irradiated cases: site, histology, margins, perineural invasion, nodal status, and indication Margin status: positive = neoplasm at margin, close = <1–2 mm from margin Nodal status: N0 = no nodal involvement, N1 = nodal metastasis

Case	Neoplasm site	Histology	Margin status	Perineural invasion	Nodal status	Indication for radiotherapy
1	Submandibular gland	Mucoepidermoid carcinoma (high-grade)	Positive	Present	N0	High-grade, positive margin, perineural invasion
2	Hard palate	Mucoepidermoid carcinoma (high-grade)	Close	Absent	N0	High-grade, close margin
3	Submandibular gland	Adenoid cystic carcinoma	Close	Present	N0	Perineural invasion, close margin
4	Parotid gland	Salivary duct carcinoma	Positive	Present	N1	High-grade, positive margin, nodal involvement
5	Submandibular gland	Squamous cell carcinoma	Positive	Absent	N0	Positive margin
6	Submandibular gland	Squamous cell carcinoma	Close	Absent	N0	Close margin
7	Submandibular gland	Mucoepidermoid carcinoma (high-grade)	Positive	Absent	N0	High-grade, positive margin
8	Parotid gland	Lymphoepithelial carcinoma	Negative	Present	N0	Perineural invasion
9	Submandibular gland	Adenoid cystic carcinoma	Negative	Present	N0	Perineural invasion
10	Maxillary sinus	Adenoid cystic carcinoma	Negative	Present	N0	Perineural invasion
11	Maxillary sinus	Adenoid cystic carcinoma	Close	Absent	N0	Close margin
12	Submandibular gland	Mucoepidermoid carcinoma (high-grade)	Positive	Absent	N0	High-grade, positive margin

Table 6 demonstrates that postoperative complications were procedure-specific, with facial nerve-related complications occurring after parotidectomy, marginal mandibular nerve injury after submandibular gland excision, and no complications following wide local excision or excision of neoplasms.

**Table 6:** Postoperative complications stratified by surgical procedure n = number of cases

Surgical procedure	Total cases (n)	Specific complication	n (%)
Superficial parotidectomy	21	Transient facial nerve weakness	2 (9.5%)
		Seroma	1 (4.8%)
Total parotidectomy	5	Persistent facial nerve weakness at three months	1 (20.0%)
Submandibular gland excision	17	Marginal mandibular nerve injury	2 (11.8%)
Wide local excision of the lesion	4	None	0
Excision of neoplasm	3	None	0
Overall	50	Any complication	6 (12.0%)

## Discussion

The peak incidence of benign neoplasms in the 5th decade (26.32%), followed by the 6th decade (18.42%), likely reflects their slow growth and prolonged subclinical course, which can delay detection until later in adulthood. Malignant neoplasms, however, were reported equally in the 4th to 6th decades (33.33%), consistent with their more aggressive biological behaviour and earlier symptom manifestation. A combination of age-related genetic changes and cumulative environmental or occupational exposures, such as tobacco use and industrial pollutants, may further explain the broader age distribution observed for malignant neoplasms. Similar findings were reported in a study by Subhashraj, where the peak age of incidence for benign neoplasms was in the 5th decade (22%) and for malignant neoplasms, the 6th decade (25%) [27]. In contrast to our study, Shrestha et al. observed the highest incidences of benign and malignant neoplasms in the 4th and 5th decades, respectively [28].

Histologically, pleomorphic adenoma and mucoepidermoid carcinoma most commonly presented in the 5th decade, whereas adenoid cystic carcinoma was frequently reported in the 4th decade; although mucoepidermoid carcinoma is often reported in pediatric populations, its predominance in adults in our cohort likely reflects regional or population-specific variation. Fomete et al. reported similar findings of pleomorphic adenoma and mucoepidermoid carcinoma, most commonly presenting in the 5th decade [11]. While de Oliveira et al. observed pleomorphic adenoma and mucoepidermoid carcinoma, mostly reported in the 4th decade [16]. In the 6th decade, Fomete et al. and de Oliveira et al. reported the highest incidence of adenoid cystic carcinoma.

Our study demonstrated a female predominance among benign neoplasms and a male predominance among malignant neoplasms. Although statistical testing was performed, the small malignant subgroup limited analytical power; thus, these observations should be interpreted as descriptive trends and not generalised beyond this study population. Similar patterns were reported by Atarbashi Moghadam et al. [29]. In contrast, Masanja et al. observed male predominance in benign neoplasms [19] and Sandhu et al. reported female predominance among malignant neoplasms [30].

Pleomorphic adenoma occurred more frequently in females in our data, consistent with the report by Trenkić Božinović et al. [7], although Pachori et al. reported more male cases [31]. Both Trenkić Božinović et al. and our studies revealed a male majority in mucoepidermoid carcinoma. In contrast, Mejía-Velázquez et al. reported female predominance [32]. These findings demonstrate variability across studies and likely reflect population-specific factors rather than consistent biological predilection. We found no evidence of a gender preponderance in adenoid cystic carcinomas. Similar results were observed by Mejía-Velázquez et al. Contrary to these findings, de Oliveira et al. reported female predominance [16], while Trenkić Božinović et al. reported male predominance.

Anatomically, pleomorphic adenoma was most commonly reported in the parotid gland, in agreement with Subhashraj and Jaafari-Ashkavandi et al. [27,33]. Mucoepidermoid carcinoma was more often located in the submandibular gland in our cohort, while Jaafari-Ashkavandi et al. reported that both pleomorphic adenoma and adenoid cystic carcinoma were frequently identified in the submandibular gland.

In every case, swelling was the primary clinical feature. Similar findings were reported by Fomete et al. [11]. For pleomorphic adenoma and mucoepidermoid carcinoma, the diagnostic accuracies were 86% and 98%, respectively. Sarkar et al. observed diagnostic accuracies of 95% and 72.7% for pleomorphic adenoma and mucoepidermoid carcinoma, respectively [34].

Surgery was the main modality of treatment, with superficial parotidectomy being the most often carried out surgical technique. This observation is similar to that of Taiwo et al. and Thiagarajan et al. [35,36]. We detected facial nerve paralysis as the most common complication, a finding that parallels earlier studies by Sathish Babu et al. and Taiwo et al. [3,35].

Limitations of the study

This study was conducted at a single centre with a relatively small sample size, which limits the statistical power and generalizability of the findings. The short follow-up period of three months precludes assessment of long-term outcomes, including recurrence rates and prolonged functional sequelae such as facial nerve recovery and salivary function. Additionally, the small number of malignant cases reduces the reliability of inferential analyses, and FNAC, while useful, carries an inherent risk of diagnostic misclassification, particularly in less common neoplasms. Molecular and immunohistochemical analyses were not performed; their inclusion could have enhanced diagnostic precision, provided additional prognostic information, and guided tailored management strategies, particularly in malignant or atypical cases. These methodological constraints should be considered when interpreting the results, and caution is warranted in extrapolating the findings to broader populations.

## Conclusions

This was a single-institution experience where analysis of 50 salivary gland neoplasms was carried out. They were predominantly observed in the 5th decade, with a female predominance overall. Females developed more benign neoplasms than males. Pleomorphic adenoma was often observed in the parotid gland, and mucoepidermoid and adenoid cystic carcinomas were frequently noticed in the submandibular gland. The most typical clinical manifestation was swelling. FNAC demonstrated moderate concordance with histopathology and remains a useful, minimally invasive diagnostic tool, although limitations exist in less common neoplasms. Surgical excision remains the mainstay of treatment, with adjuvant radiotherapy used for malignant cases, and complications are largely procedure-specific, including transient facial nerve or marginal mandibular nerve injury. These results reflect our centre’s experience, and further multicentre studies with larger sample sizes and extended follow-up are needed to characterise these neoplasms more accurately.
